# Medium-chain acyl-CoA dehydrogenase deficiency associated with a novel splice mutation in the *ACADM* gene missed by newborn screening

**DOI:** 10.1186/s12881-015-0199-5

**Published:** 2015-07-30

**Authors:** Sarah C. Grünert, A. Wehrle, P. Villavicencio-Lorini, E. Lausch, B. Vetter, K. O. Schwab, S. Tucci, U. Spiekerkoetter

**Affiliations:** Center of Pediatrics and Adolescent Medicine, University Hospital Freiburg, Mathildenstraße 1, 79106 Freiburg, Germany; Present address: Department of Human Genetics, Halle University Hospital, Ernst-Grube-Str. 30, 06097 Halle, Germany; Römerstrasse 38, 79423 Heitersheim, Germany

**Keywords:** *ACADM*, Missplicing, Newborn screening, False negative

## Abstract

**Background:**

Medium-chain acyl-CoA dehydrogenase (MCAD) deficiency is the most common disorder of mitochondrial fatty acid β-oxidation and a target disease of newborn screening in many countries.

**Case presentation:**

We report on two siblings with mild MCAD deficiency associated with a novel splice site mutation in the *ACADM* gene. The younger sibling was detected by newborn screening, while the older sister was missed, but diagnosed later on by genetic family testing. Both children were found to be compound heterozygous for the common c.985A > G (p.K329E) mutation and a novel splice site mutation, c.600-18G > A, in the *ACADM* gene. To determine the biological consequence of the c.600-18G > A mutation putative missplicing was investigated at RNA level in granulocytes and monocytes of one of the patients. The splice site mutation was shown to lead to partial missplicing of the *ACADM* pre-mRNA. Of three detected transcripts two result in truncated, non-functional MCAD proteins as reflected by the reduced octanoyl-CoA oxidation rate in both patients. In one patient a decrease of the octanoyl-CoA oxidation rate was found during a febrile infection indicating that missplicing may be temperature-sensitive.

**Conclusions:**

Our data indicate that the c.600-18G > A variant activates a cryptic splice site, which competes with the natural splice site. Due to only partial missplicing sufficient functional MCAD protein remains to result in mild MCADD that may be missed by newborn screening.

**Electronic supplementary material:**

The online version of this article (doi:10.1186/s12881-015-0199-5) contains supplementary material, which is available to authorized users.

## Background

Medium-chain acyl-CoA dehydrogenase deficiency (MCADD; MIM 201450) is an autosomal recessive disorder of mitochondrial β-oxidation, caused by mutations in the *ACADM* gene. The estimated prevalence of MCADD from newborn screening ranges from 1 in 8,100 to 1 in 27,000 among populations of mostly European descent and is less common in populations of non-European origin [1]. Clinical manifestation comprises hypoketotic hypoglycemia, muscular hypotonia, lethargy, vomiting, seizures, encephalopathy, coma, and death. Since fatty acid oxidation is essential for energy production during fasting and increased energy expenditure, metabolic decompensations are predominantly precipitated by metabolic stress. In the absence of screening, mortality is up to 26 % [2] and many surviving patients develop severe neurocognitive impairment [1–5]. Since the implementation of tandem mass spectrometry-based newborn screening (NBS) the prognosis of this inherited metabolic disease has significantly improved [6–8]. However, NBS also identifies mild and potentially asymptomatic cases and sometimes also heterozygous individuals, who are at no risk of clinical symptoms.

The typical MCADD acylcarnitine pattern includes elevated levels of C6, C8, C10 and C10:1 acylcarnitines as well as increased disease-specific acylcarnitine ratios [C8]/[C10], [C8]/[C12] and [C8]/[C2] [9]. Additionally, elevated levels of hexanoylglycine, isohexanoylglycine, suberylglycine, and phenylpropionylglycine may be found in the urine of affected individuals. Enzyme activity of MCAD can be determined by measurement of octanoyl-CoA or phenylpropionyl-CoA oxidation rate in lymphocytes and may be used for risk assessment [10]. This is of special importance as the molecular diagnostic after a suggestive NBS result often leads to the identification of novel DNA variants, or “variants of unknown significance” [11].

We report on two asymptomatic siblings with a biochemically mild MCADD phenotype (low levels of MCAD-typical metabolites and high residual enzyme activity). Only one of them was detected by NBS, while the other one was missed. Mutation analysis revealed a known pathogenic missense mutation in combination with a novel splice site mutation in the *ACADM* gene. The consequences of this splice site mutation were further characterized.

## Patients

### Consent

All individuals in our study were recruited by physician-initiated referral. The study was conducted in accordance with the Declaration of Helsinki protocols and approved by the institutional ethics review board of Freiburg University Hospital, Germany. Written informed consent for molecular studies was obtained from the affected individuals and/or their legal guardians in accordance with current German law (GenDG). A copy of the written consent is available for review by the Editor of this journal. Control samples and primary cells were collected from ancestry-, sex- and age-matched healthy individuals under the same criteria and regulations.

## Case presentations

Patient 1, a 7-year-old girl, is the first child of non-consanguineous German parents. She was born spontaneously at 41 weeks of gestation after an uneventful pregnancy. Birth weight, birth length and head circumference were 3280 g (56th percentile), 53 cm (87th percentile) and 35 cm (50th percentile), respectively. Three hours after birth she was admitted to the intensive care unit with suspected newborn infection. She was pale, tachypnoeic and required CPAP ventilation with up to 60 % oxygen. Laboratory parameters were indicative of systemic infection (Interleukin 6 727 pg/ml, reference range < 30 pg/ml) and chest X-ray revealed pneumonic infiltrations. Under antibiotic treatment her general condition ameliorated quickly, and the child was dismissed from hospital on day 10. NBS results including acylcarnitine analysis were unremarkable. After dismission from hospital no further problems occurred and the child developed normally.

**Fig. 1:**
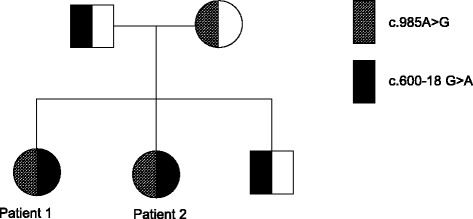
Pedigree of the patient’s family showing haplotypes for the *ACADM* allele variants in the patient, her siblings and her parents. Males are presented by squares, females by circles. Allele variants without mutation are represented by an open square or circle. Mutated allele variants are indicated by a solid (c.600-18G > A) or a shaded (c.985A > G) square or circle, respectively

Five years later her younger sister, patient 2, was born and NBS was suggestive of MCADD. Urinary organic acid analysis biochemically confirmed the diagnosis. Mutation analysis revealed compound heterozygosity for the common missense mutation c.985A > G and an intronic sequence variant, c.600-18G > A, which has not been described previously. Genetic family screening was performed showing that the older sister (patient 1) was also compound heterozygous for the same two mutations. Heterozygosity for the c.985A > G (p.K329E) mutation was detected in the healthy mother, while the healthy father and the healthy 3-year-old brother were found to be heterozygous for the intronic sequence variant. An overview on the *ACADM* genotypes of the two patients, their brother and their parents is shown in Fig. 1.

In the following 3 years acylcarnitine analyses in dried blood spots of both girls were performed repeatedly at times of physical well-being and yielded variable results (Table 1): In patient 1 three of four samples showed an unremarkable acylcarnitine pattern while patient 2 usually displayed a “typical” MCAD pattern. Free carnitine concentrations were always within the normal range. Analysis of organic acids in urine showed only mildly elevated concentrations of hexanoylglycine and isohexanoylglycine in both patients (Table 1). Suberylglycine was only detected in few samples and the concentration of dicarboxylic acids was usually not elevated. The oxidation rate of octanoyl-CoA was measured in lymphocytes of both patients. In patient 1 enzyme testing was performed during a febrile episode yielding 17 % of healthy controls. In patient 2, octanoyl-CoA oxidation rate was determined twice, at time of physical well-being and during fever showing a residual activity of 24 % and 13 %, respectively (Table 1).

**Table 1:** Biochemical data of two siblings with mild MCAD deficiency

		Patient 1	Patient 2	Controls
Newborn screening		Unremarkable	Suggestive of MCADD	Unremarkable
Confirmatory testing				
Acylcarnitines in dried blood spots (at different time points)	C6	0.06–0.27 μmol/l	0.13–0.38 μmol/l	<1 μmol/l
	C8	0.11–1.56 μmol/l	0.31–1.5 μmol/l	<0.4 μmol/l
	C10 (including C4:1-DC)	0.07–0.74 μmol/l	0.16–0.93 μmol/l	<0.2 μmol/l
	C10:1	0.07–0.63 μmol/l	0.13–0.7 μmol/l	<0.2 μmol/l
	[C8]/[C10]	1.2–2.46	1.62–2.86	<5
	[C8]/[C12]	4.97–19.3	6.46–17.1	<5
	[C8]/[C2]	0.01–0.09	0.02–0.4	<0.02
Urinary organic acids (at different time points)	Hexanoylglycine	+/ n.d.	+/ n.d.	n.d.
	Isohexanoylglycine	+/ n.d.	+/ n.d.	n.d.
	Suberylglycine	+/ n.d.	+/ n.d.	n.d.
	Dicarboxylic acids	Not elevated	Not elevated	
Enzyme activity measurements in lymphocytes	Octanoyl-CoA-oxidation	17 % (during fever)	24 % (without fever)	100 %
			13 % (during fever)	

*n.d.* not detectable, + detectable in traces

No inborn errors of metabolism had been detected in other family members so far, and no cases of unexplained death in infancy were reported. When specifically asked, the parents reported that the older sister (patient 1) had well tolerated overnight-fasts of 9–10 h at the age of 9 months. She had had several minor febrile infections including gastroenteritis within her first year of life. At the age of 4.5 years she had suffered from a severe febrile gastroenteritis. During this episode she ate and drank only sparingly for about 4 to 5 days. Despite this catabolic state her vigilance had always been normal with no signs of somnolence or lethargy and clinical symptoms of hypoglycemia were not observed.

## Materials and methods

### Oxidation rate of medium-chain acyl-CoAs

Determination of the MCAD residual activity was achieved by assaying the oxidation of octanoyl-CoA and C10-CoA in lymphocytes by HPLC as described previously [10].

### Mutation analysis

Genomic DNA was extracted from EDTA blood with QIAmp® DNA Mini Kit (Qiagen, Hilden, Germany). All exons and parts of the flanking intronic regions of the *ACADM* gene were amplified according to standard protocols using Quick-Load® Taq 2X Master Mix (New England Biolabs, Frankfurt, Germany). Sequencing reactions were performed with the BigDye® Terminator v3.1 Cycle Sequencing Kit (Applied Biosystems, Weiterstadt, Germany) (primer sequences are available on request) and were analysed using a 3730xl DNA Analyzer (Applied Biosystems, USA). Electropherograms were evaluated with the Sequencing Analysis Software v5.4 (Applied Biosystems, USA). To predict a putative missplicing effect of the sequence variant c.600-18G > A the Alamut software 2.3.2 was used.

### Definition of RNA splicing patterns

Peripheral blood samples were fractionated into mononuclear cells and granulocytes using the standard Ficoll protocol. Total RNA was isolated from these cells using TRIzol® Reagent (Life Technologies, USA) followed by a phenol-chloroform extraction, treatment with DNAse I (Roche, Mannheim, Germany) and purification with the RNeasy Mini Kit (Quiagen, Hilden, Germany). Reverse transcription of RNA into cDNA with oligo-dT primers and M-MLV RT (Life Technologies, USA) was conducted according to the manufacturer’s protocol. The subsequent PCR amplification was performed with specific primers spanning from exons 7 to 9 of the *ACADM* gene (For_AACCTGGAGCAGGCTCTGAT and Rev_ACCAGCTCCGTCACCAATTA). PCR products were detected by electrophoresis on a 2 % agarose gel. After excision and gel extraction with the QIAquick Gel Extraction Kit (Qiagen, Hilden, Germany), PCR products were evaluated by sequencing analysis (3130xl Genetic Analyzer, Applied Biosystems, USA). Comparative alignments with the reference coding sequence were carried out by the DNA Sequence Analysis Software Sequencher 4.9 (Gene Codes Corporation, USA).

PCR products of the patient’s monocytes cDNA were ligated into the pCR™II Vector and transformed into TOP10F’ Chemically Competent *E. coli* with the TA Cloning® Kit (Life Technologies, USA), according to the manufacturer’s protocol. Plasmids containing the different PCR products were purified with the FastPlasmid Mini Kit (5 Prime, Hamburg, Germany). After an *Eco*RI digestion (New England Biolabs, Frankfurt, Germany) fragments were detected on an agarose gel and different splice variants were identified by their predicted size. To confirm the proportion of the different splicing variants gel fragments were excised, extracted and sequenced as described above.

Fluorescent fragments of DNA were generated via PCR amplification of the cDNA gained from granulocytes and monocytes of the patient and two independent controls with the For_AACCTGGAGCAGGCTCTGAT primer and a Rev_ACCAGCTCCGTCACCAATTA 5′ FAM labelled primer, respectively. Fragments were separated using capillary electrophoresis (3130xl Genetic Analyzer, Applied Biosystems, USA) and sized by comparison to a size standard (GeneScan™ 500 LIZ® Size Standard, Life Technologies, USA).

## Results

In the Single Nucleotide Polymorphism database (dbSNP) the sequence variant c.600-18G > A is annotated as an intronic variant with unknown clinical relevance. *In silico* splice prediction with the Alamut Interactive Biosoftware was performed and all tools integrated in this application (Splice Site Finder (SSF), MaxEntScan (MES), Neural Network (NNSplice) and GeneSplicer) identified the variant as a possible candidate for splicing alteration (Additional file 1: Table S1).

**Fig. 2:**
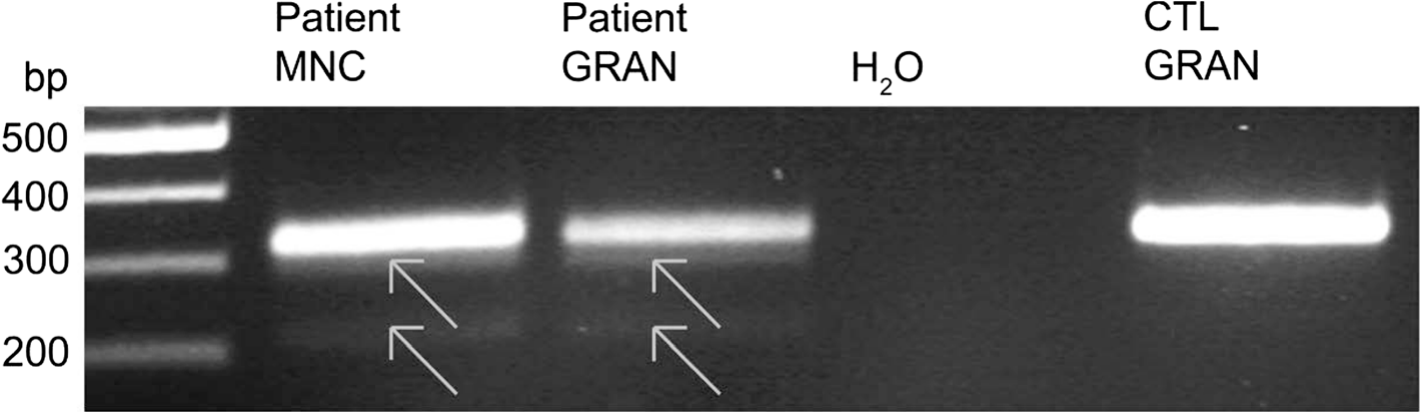
A novel mutation in the *ACADM* gene results in aberrant splicing. Gel documentation visualizes the PCR product of a region of 323 bp from exon 7 to 9 of the *ACADM* gene in cDNA of control granulocytes (CTL GRAN) as well as of monocytes (MNC) and granulocytes (GRAN) of patient 1. Arrows point to the additional PCR products that were detected using cDNA of the patient’s granulocytes and monocytes. Analysis of the two additional PCR products in the gel eluates of the patient’s samples revealed missplicing resulting in either a partial loss of exon 8 (fragment of 294 bp, upper arrowhead) or complete skipping of exon 8 (fragment of 215 bp, lower arrowhead)

To determine the biological consequence of the c.600-18G > A mutation putative missplicing was investigated at RNA level in granulocytes and monocytes of patient 1. Besides the expected PCR products of ca. 323 bp, we observed additional PCR products of 215 and 294 bp which were only present in the patient’s sample (Fig. 2). Gel extraction followed by sequencing revealed either a total (215 bp) or partial deletion (294 bp) of exon 8.

**Fig. 3:**
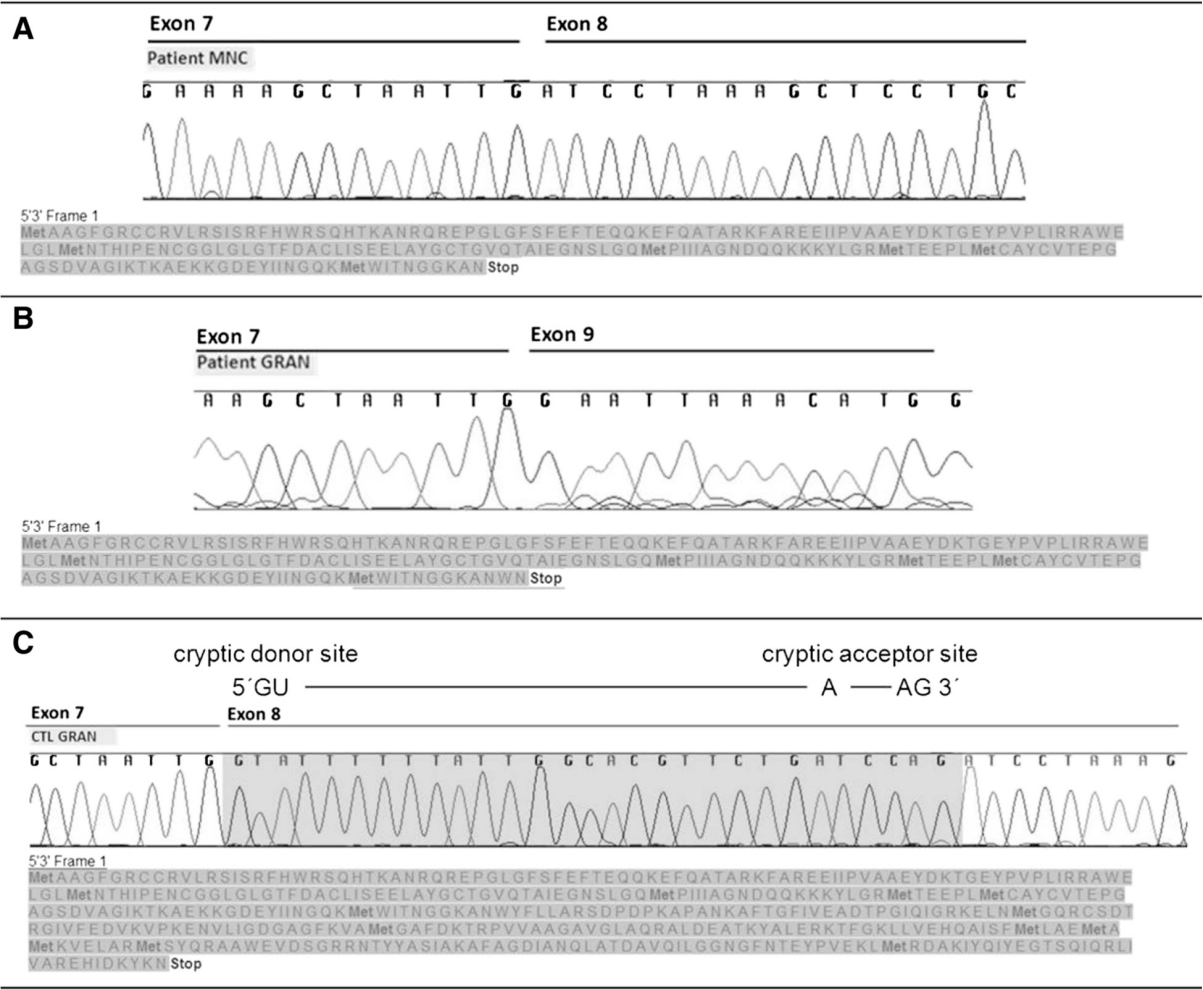
Missplicing leads to premature stop codons and therefore truncated MCAD protein products. Shown are extracts of chromatograms representative for the different splice variants. Specific regions are indicated (exon 7 to exon 9). *In silico* protein products of the *ACADM* gene were generated, the amino acid sequence is displayed under the chromatogram extracts. Both forms of missplicing lead to premature stop codons and therefore truncated protein products which only differ in two amino acids (WN) at the C-terminus of the truncated protein (**a** and **b** corresponding to fragments 294 bp and 215 bp, respectively). **c** The region highlighted in grey in the chromatogram reflects the region that is partly deleted in the patient’s cDNA shown in A due to missplicing. Probable cryptic donor and acceptor sites that lead to missplicing are indicated. (CTL GRAN = control granulocytes, MNC = monocytes GRAN = granulocytes)

In silico translation of both splice variants with the help of the ExPASy translate tool predicts premature stop in the patient’s variants due to missplicing and therefore truncated proteins leading most likely to dysfunctional enzyme (Fig. 3).

**Fig. 4:**
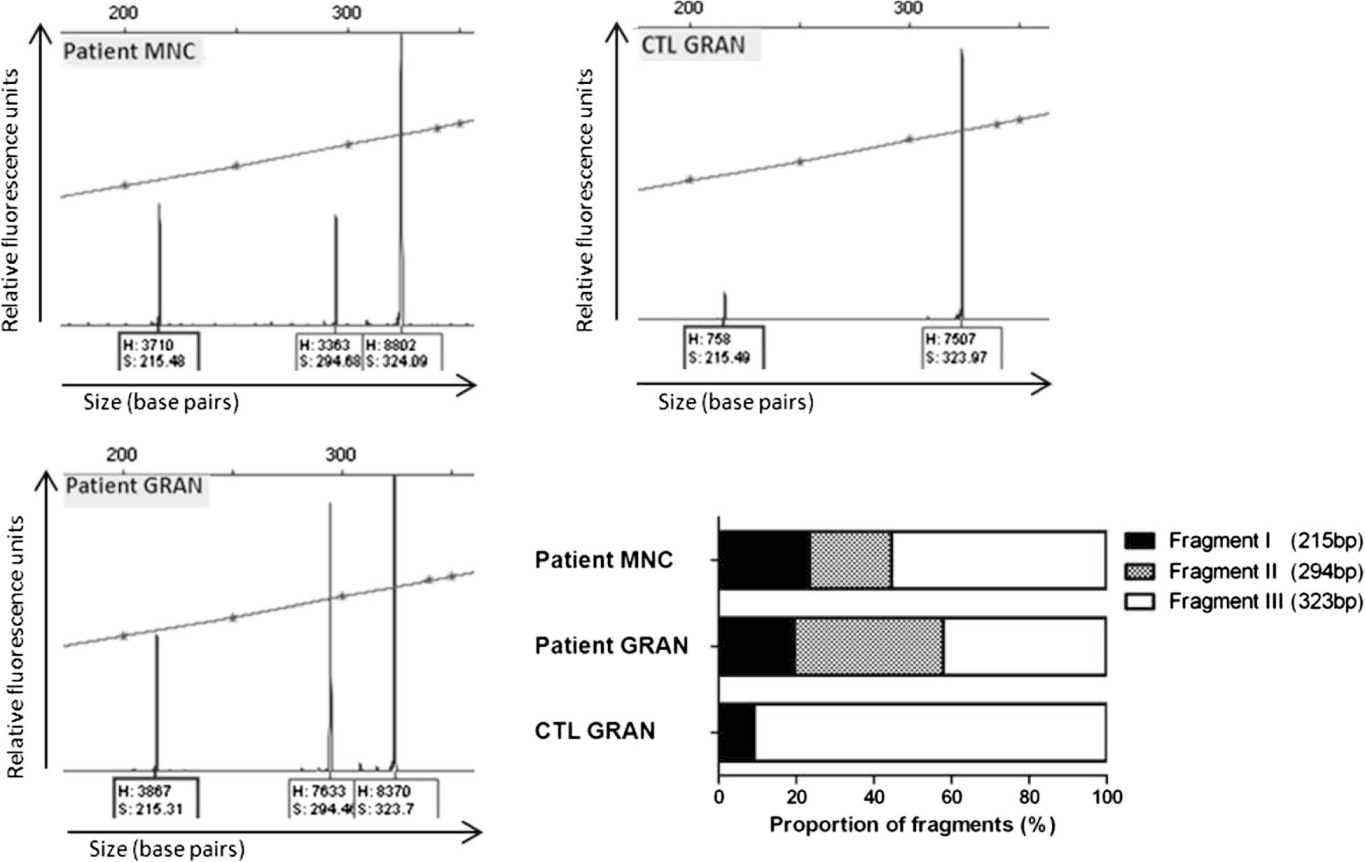
Percental amounts of different splice products (studied in patient 1). Previously identified splicing fragments were confirmed with fragment analysis (H = height, S = size of the fragment). Data of the fragment analysis were tabularized (Additional file 3: Table S2) and single fragment heights were set in relation to overall heights (summarized fragment heights of the particular sample) to determine the percental distribution of the different splice products in each sample. (CTL GRAN = control granulocytes, MNC = monocytes, GRAN = granulocytes)

To quantify missplicing two different approaches were used. In the first approach, cDNA of the patient’s monocytes was cloned into the pCRII-TOPO vector. With this method, the different RNA splicing variants could be identified by their calculated transcript lengths. The different splice variants were further confirmed by sequencing. The evaluation of 28 transcripts revealed missplicing in half of all PCR products: Besides the 14 wild type sequences, 5 sequences (18 %) showed skipping of exon 8, and in 9 sequences (32 %) partial deletion of exon 8 could be confirmed (Additional file 2: Figure S1). Consistent with this finding, the second quantitation approach, based on a fragment analysis with a 5′ reverse FAM labelled primer, revealed an approximate ratio of 45:55 of aberrant splicing *versus* normal splicing in the patient’s monocytes and an approximate ratio of 60:40 in the patient’s granulocytes, respectively (Fig. 4, Additional file 3: Table S2). Interestingly, a minor amount (~10 %) of the transcript with exon 8 skipping could also be detected in the control sample. Therefore, it is conceivable that ‘natural’ missplicing of the *ACADM* RNA also occurs in healthy individuals although to a very limited extent as we could not detect this transcript in the gel.

**Fig. 5:**
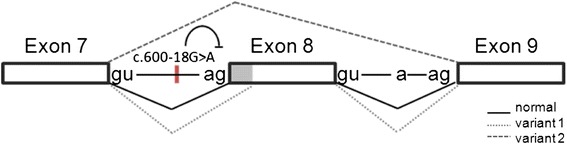
Working model of the splice reaction with cryptic donor and acceptor sites. Exons are boxed, introns are represented by lines. The mutation in intron 7 of the patient leads to the activation of cryptic donor and acceptor sites that compete with the natural splice site thereby resulting in partial deletion (variant 1) or skipping of exon 8 (variant 2)

In a last step, the transcript sequences were scanned for possible cryptic splice sites and a scheme for the aberrant splicing process could be developed. In our model, the c.600-18G > A mutation in intron 7 leads to a splice pattern with strong cryptic donor and acceptor sites that compete with the natural splice sites, resulting either in partial deletion or complete skipping of exon 8 (Fig. 5). We, therefore, presume the sequence variant c.600-18G > A to be likely pathogenic which is also reflected by the reduced octanoyl-CoA oxidation rate in both patients.

## Discussion

We here report on two siblings with mild MCADD, of whom only one was identified by newborn screening. The genotypes of patients identified after implementation of NBS differ from those identified in patients with clinical manifestation [12, 13]. The mutation c.985A > G is the most common mutation in clinically detected patients and has been found in 80 % of these individuals in homozygosity and in 18 % in heterozygosity [14, 15]. This mutation is considerably less frequent in individuals diagnosed by NBS, while numerous novel mutations including a second prevalent mutation, c.199 T > C, have been identified in this group of patients [12, 13]. Individuals carrying these mutations often show significantly lower concentrations of disease-characteristic acylcarnitines [13, 14]. Our patients were found to be compound heterozygous for the common c.985A > G mutation and a novel splice site mutation, c.600-18G > A. The c.985A > G mutation (K329E) is well characterized with respect to its functional consequences. The MCAD protein is a tetramer of 4 identical subunits. The amino acid change K329E is located in the C terminal α domain of the enzyme and compromises tetramer assembly by affecting helix-helix interactions resulting in degradation of the mutant enzyme [16]. The K329E protein variant has been shown to be thermosensitive [17]. Therefore, febrile episodes may additionally affect protein stability and function in patients carrying this mutation and may aggravate energy deficiency [18].

The c.600-18G > A mutation has not been described previously. Variant analysis using a prediction software indicated a splice altering mutation. Indeed, we could demonstrate the pathogenicity of this mutation by proof of aberrant splicing. We propose an activation of cryptic splice sites that leads to weakening of the natural splice sites and generates shortened transcripts. Although missplicing is not complete, the combination of both mutations in the compound heterozygous state may account for the reduced enzyme activity and biochemical abnormalities in our patients. The residual enzyme activity measured with octanoyl-CoA as substrate was 13-24 % of healthy controls. These values are higher than those usually found in patients with “classical” MCADD, but lower than those found in heterozygous carriers [19] indicating that the splice site mutation really has a compromising effect on the MCAD protein. Missplicing is known to be highly tissue and cell type specific as can be seen from the different splicing patterns in granulocytes and monocytes of our patients. Though no prediction of the prevalence of missplicing in hepatocytes can be made from our data, it is conceivable that there is a shift in favour of the aberrant splice product in this cell type. Furthermore, it has been reported that splicing processes can be temperature-sensitive. In case that the original splice site is still functional temperature may affect splice site selection in favour of the aberrant splice site [20, 21]. Interestingly, we found a lower residual activity during a febrile infection than at time of physical well-being in patient 2 (Table 1). In the context of MCADD, in which metabolic decompensations are often triggered by febrile infections, this may be of clinical relevance. Enhanced missplicing during episodes of increased energy expenditure may aggravate the cellular energy deficiency and therefore have clinical consequences.

Only one of the two sisters was identified by newborn screening due to a characteristic acylcarnitine pattern. This is notable as it has been shown that even carriers of a single c.985A > G mutation may display mildly elevated C8 acylcarnitine concentrations [22, 23]. Lehotay et al. reported significantly higher C8 concentrations of 0.302 ± 0.09 μmol/l in c.985A > G heterozygotes compared to healthy controls, while the C8 concentration in our compound heterozygous patient (0.17 μmol/l) was even below this range. High C8 levels have been shown to be associated with severe mutations including the common c.985G > C mutation (in homozygosity), deletions, nonsense or splice site mutations [24]. In a study with 34 MCAD deficient individuals evaluating the correlation between genotype and biochemical phenotype, patients homozygous for c.985A > G had the highest levels of neonatal octanoylcarnitine followed by compound heterozygotes of c.985A > G and other mutations. Patients without c.985A > G and compound heterozygotes for c.985A > G and c.199 T > C had the lowest levels of C8. According to this, an intermediate elevation of the C8 acylcarnitine concentration would have been expected in our patient.

Even if the high residual MCAD activity and the mild biochemical expression in our patients are suggestive of a presumably low clinical penetrance, the potential risk of metabolic decompensation during severe catabolism cannot be estimated and the clinical relevance of mild MCADD is difficult to establish. Although the majority of symptomatic patients present within the first three years of life, several late onset cases have been reported [25, 26]. Both girls have remained asymptomatic so far, however, they are only 3 and 7 years old, respectively. Nevertheless, it is of great interest that patient 1 has experienced many infections and catabolic conditions before diagnosis by family screening not resulting in any clinical symptoms. The natural course of the disease in individuals with novel mutations is generally unknown [16]. Many of these children may remain asymptomatic even without detection by NBS. This is reflected by the much higher detection rate of MCADD by NBS compared to clinically detected cases in unscreened populations. In contrast, one more recent study on structural alterations of several mutant variants proposed that novel mutations found in NBS, including the c.199 T > C mutation, do not bear a lower risk of metabolic decompensation than that associated with mutations detected in clinically ascertained patients [16], although, to our knowledge, only one individual carrying the c.199 T > C mutation together with a second mutation has ever been symptomatic during follow-up. The mother sought emergency room care for the infant two times in the first year for listlessness associated with gastroenteritis. At age 22 months altered mental status and variable hypo- and hyperglycemia were reported during hospital admission for hand/foot/mouth disease [24]. Residual enzyme activities in patients with proposed mild MCADD are in some cases in the same activity range as in definite heterozygous parents questioning the clinical relevance of the mutations [10]. So far, there is no consensus to consider certain *ACADM* genotypes as safe and protective from clinical symptoms [16, 24], however, we are also aware of an overtreatment of many individuals identified by newborn screening. The same uncertainty is true with respect to the interpretation of the biochemical phenotype despite a strong relationship between initial C8 levels and outcome [24].

## Conclusion

The novel splice variant c.600-18G > A was shown to activate a cryptic splice site, which competes with the natural splice site. Due to only partial missplicing sufficient functional MCAD protein remains to result in clinically and biochemically mild MCADD that may be missed by newborn screening. If patients with such mild phenotypes are at risk of fatal metabolic decompensations remains a matter of debate.

## Additional files

Additional file 1: Table S1.
*In silico* splice prediction of the Alamut Interactive Biosoftware. Note that all tools integrated in this application (Splice Site Finder (SSF), MaxEntScan (MES), Neural Network (NNSplice) and GeneSplicer) identified the variant as a possible candidate for splicing alteration. Additional file 2: Figure S1. Gel documentation of PCR products of the patient’s monocyte cDNA after *Eco*RI digestion. Fragments were detected on an agarose gel. Splice variants were identified by their predicted size and confirmed by sequence analysis. Missplicing was detected in half of the fragments. Additional file 3: Table S2. Tabularized data of the fragment analysis. Different fragments are indicated with I (215 bp), II (294 bp) or III (323 bp). Single fragment heights were set in relation to overall heights (summarized fragment heights of the particular sample) to determine the percental distribution of the different splice products in each sample. CTL GRAN = control garanulocytes, Patient GRAN = granulocytes of patient, Patient MNC = monocytes of patients.

## Abbreviations

C8: Octanoyl-carnitine
MS/MS: Tandem mass spectrometry
MCAD: Medium chain acyl-CoA dehydrogenase
MCADD: Medium chain acyl-CoA dehydrogenase deficiency
NBS: Newborn screening
