# Supplementary figures and images for: Medium-chain acyl-CoA dehydrogenase deficiency associated with a novel splice mutation in the ACADM gene missed by newborn screening
Source: BMC Med Genet. 2015 Jul 30;16:56. doi: 10.1186/s12881-015-0199-5 (PMC4557819; doi:10.1186/s12881-015-0199-5)

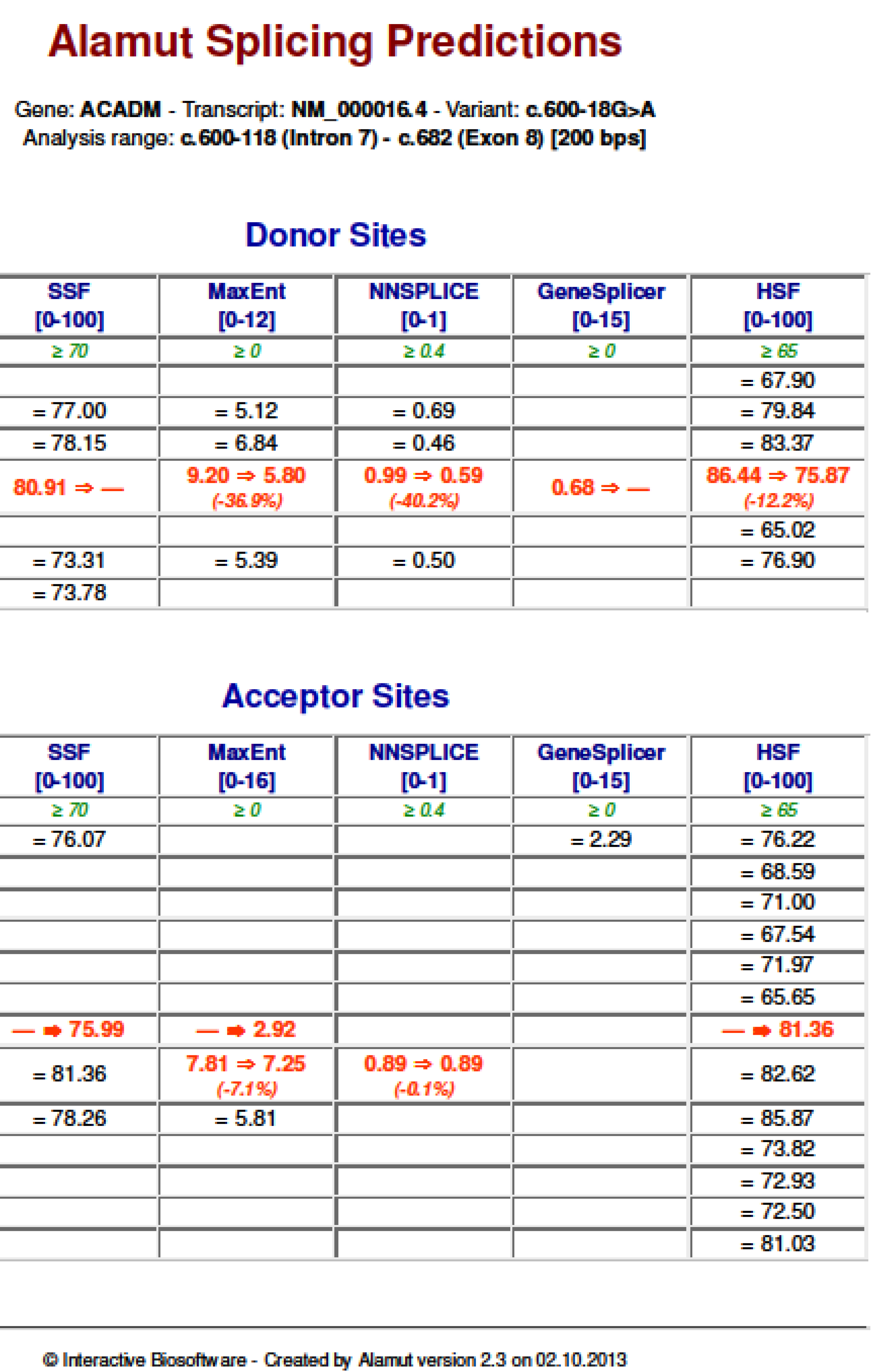

Supplement: Additional file 1: Table S1. — In silico splice prediction of the Alamut Interactive Biosoftware. Note that all tools integrated in this application (Splice Site Finder (SSF), MaxEntScan (MES), Neural Network (NNSplice) and GeneSplicer) identified the variant as a possible candidate for splicing alteration. [file 12881_2015_199_MOESM1_ESM.doc]

## Slide 1
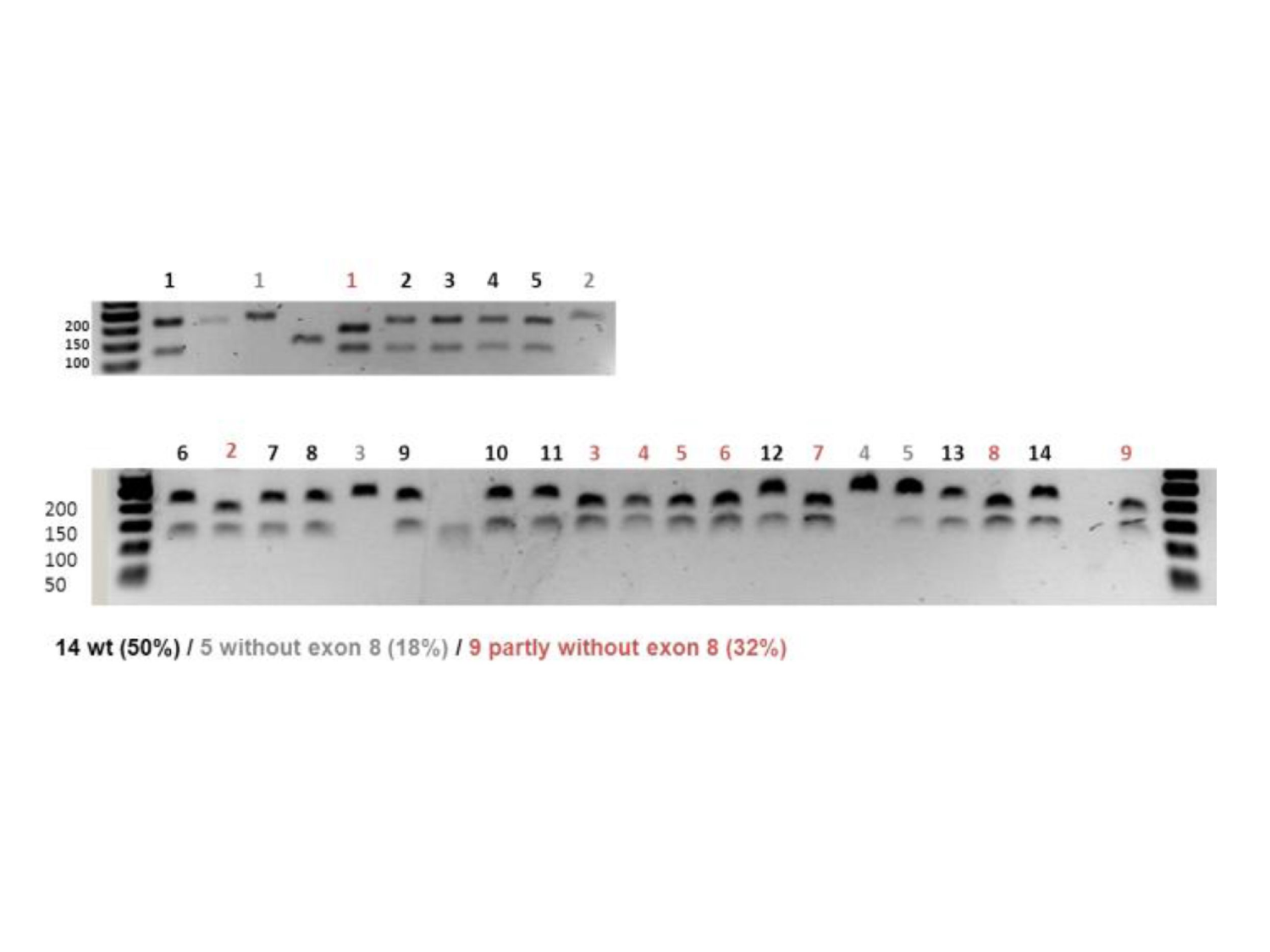

Supplement: Additional file 2: Figure S1. — Gel documentation of PCR products of the patient’s monocyte cDNA after EcoRI digestion. Fragments were detected on an agarose gel. Splice variants were identified by their predicted size and confirmed by sequence analysis. Missplicing was detected in half of the fragments. [file 12881_2015_199_MOESM2_ESM.ppt]

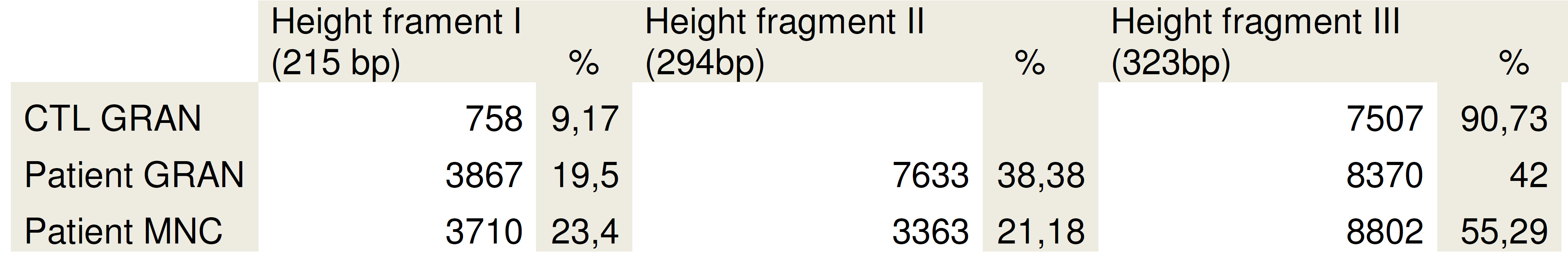

Supplement: Additional file 3: Table S2. — Tabularized data of the fragment analysis. Different fragments are indicated with I (215 bp), II (294 bp) or III (323 bp). Single fragment heights were set in relation to overall heights (summarized fragment heights of the particular sample) to determine the percental distribution of the different splice products in each sample. CTL GRAN = control garanulocytes, Patient GRAN = granulocytes of patient, Patient MNC = monocytes of patients. [file 12881_2015_199_MOESM3_ESM.doc]
